# Increased expression of *PIN1 *gene in papillary thyroid carcinoma

**DOI:** 10.1186/1756-6614-4-4

**Published:** 2011-01-10

**Authors:** Andrzej Lewiński, Ewa Brzeziańska, Karolina Czarnecka, Joanna Latek, Włodzimierz Koptas, Anna Cyniak-Magierska

**Affiliations:** 1 Department of Endocrinology and Metabolic Diseases, Medical University of Lodz, Poland; 2 Department of Endocrinology and Metabolic Diseases, Polish Mother's Memorial Hospital - Research Institute, Lodz, Poland; 3 Department of Molecular Basis of Medicine, Medical University of Lodz, Poland; 4 Department of General and Colorectal Surgery, Medical University of Lodz, Poland

## Abstract

**Background:**

Peptidyl-prolyl *cis/trans *isomerase (Pin1), encoded by *PIN1 *gene with *locus *in chromosome 19p13, is an enzyme that catalytically induces conformational changes in proteins after phosphorylation on serine or threonine residues preceding proline (pSer/Thr-Pro motifs); in this way, it has an influence on protein interactions and intracellular localizations of proteins. The aim of the study were: 1) an assessment of *PIN1 *gene expression level in benign and malignant thyroid lesions; 2) the evaluation of possible correlations between gene expression and histopathological variants of papillary thyroid carcinoma (PTC) or tumour size, classified according to TNM classification of primary tumours (in case of PTC only); 3) the estimation of possible relationships between expression of the gene in question and patients' sex or age.

**Methods:**

Seventy (70) tissue samples were analyzed: 32 cases of PTC, 7 cases of medullary thyroid carcinoma (MTC), 7 cases of follicular adenoma (FA), and 24 cases of nodular goitre (NG). In real-time polymerase chain reaction (real-time PCR), two-step RT-PCR (reverse transcriptase-polymerase chain reaction) in an ABI PRISM 7500 Sequence Detection System was employed. The *PIN1 *gene expression level was assessed, calculating the mean relative quantification rate (RQ rate) increase for each sample.

**Results:**

The level of *PIN1 *gene expression (compared to that in macroscopically unchanged thyroid tissue) was higher in PTC group than those in FA, MTC and/or NG groups, but the statistical significance was noted for difference between PTC and NG groups only. On the other hand, the differences of RQ rate value between different PTC variants were statistically insignificant. No correlations were found between RQ values and tumour size, as well as between RQ values and patients' sex or age in PTC group.

**Conclusions:**

The *PIN1 *gene expression may have - in future - an important meaning in the diagnostics of PTC and in understanding its pathogenesis. However, our results - mostly due to the small number of cases - do not yet allow considering *PIN1 *gene as a prognostic molecular PTC marker.

## Background

The *PIN1 *gene encodes an essential peptidyl-prolyl *cis/trans *isomerase (PPIase; EC 5.2.1.8) which has a profound impact on key proteins involved in the regulation of cell cycle [1]. The human *PIN1 *gene is mapped to chromosome 19p13 [2]. Catalyzing conformational changes in substrates after phosphorylation, Pin1 recognizes phospho-serine or phospho-threonine bonds followed by proline (pSer/Thr-Pro motifs) and plays a vital role in protein folding or refolding [3]. Through isomerization Pin1 can modulate the conformation of protein substrates, changing their enzymatic activity which leads to alteration in different cell function [1].

Pin1 enzyme activity is important for oncogenic and cell-signalling pathways *via *conformational changes following phosphorylation of many molecules (e.g. Bcl-2, p53, c-Jun, beta-catenin, cyclin D1 and RAF kinase) [4-7]. The Pin1-regulated alteration in protein interactions and stability have been shown to be associated with cell transformation and progression of certain types of human tumours (e.g., prostate, colon, lung, ovary and breast cancers, and lymphoma) [8-10]. It has also been reported that inhibition of Pin1 function in human tumour cells, using Pin1 antisense RNA, induces mitotic arrest and apoptosis [11]. Further studies indicate that Pin1 plays a critical role in cell restriction points - particularly in G1/S phase; Pin1 modulates some important regulators of that cell cycle point, e.g. cyclin D1 and its upstream transcriptional factors. The increased Pin1 expression correlates significantly with overexpression of cyclin D1 mRNA and protein. Interestingly, Pin1 binds phosphorylated c-Jun, influencing the increase of its transcriptional activity towards cyclin D1 - by activating c-Jun/AP-1 and beta-catenin/T-cell factor (TCF) and, in this way it cooperates with Ras signalling pathway [4].

Pin1 is involved not only in the pathogenesis of human cancer, but - presumably - also in the pathogenesis of asthma and Alzheimer's disease [12].

Recently, in some reports - including the study on thyroid tumours - strong correlation between Pin1 and cyclin D1 immunoexpression and/or cyclin D1 mRNA and *PIN1 *expression *via *interaction with Wnt signalling pathway has been observed [13]. Moreover, it has been suggested that Pin1 may promote cyclin D1 overexpression directly or through accumulation of beta-catenin in thyroid cancer cells [13].

There are rather scarce reports which are focused on evaluation of PIN1 mRNA expression in thyroid tumours. Therefore, in order to recognize the Pin1 role in the pathogenesis of thyroid gland, we have compared the levels of *PIN1 *gene expression in benign and malignant thyroid lesions. We have assessed *PIN1 *gene expression in real-time polymerase chain reaction (real-time PCR), two-step RT-PCR (reverse transcriptase-polymerase chain reaction) and we have evaluated the possible correlations between the gene expression and the histopathological variant of examined PTCs (classic, tall-cell, follicular), tumour size grouped according to TNM classification of primary tumours (pT1, pT2, pT3, pT4). We have also decided to estimate the possible relationships between expression of the gene in question and patients' sex or age.

## Materials and methods

The Ethical Committee of the Medical University of Lodz approved the studies reported in the present article.

### Thyroid tissue samples

Tumour tissue samples (100-150 mg) were obtained from 70 patients who had undergone surgery treatment at the Centre of Oncology, the Maria Skłodowska-Curie Institute in Gliwice, at the Holy Family Municipal Hospital in Lodz and at the Department of General and Colorectal Surgery, Medical University of Lodz, Poland during the years 2002-2007. Total tumour tissue samples, immediately after resection, were collected into lysis buffer (Buffer RLT, Qiagen Sciences, USA).

Among the studied samples 32 cases of primary papillary thyroid carcinoma - PTC (26 females, 6 males; mean age 41.8 ± 15.7; mean ± SD) were used. Histopathological variants of PTC were as follows: PTC classic variant (20 cases: 17 females, 3 males; mean age 36.5 ± 14.4), PTC follicular variant (9 cases: 6 females, 3 males; mean age 49.9 ± 16.0), PTC tall-cell variant (3 cases: 3 females; mean age 53.3 ± 5.1) (Table 1). Seven (7) cases of medullary thyroid carcinoma - MTC (6 females, 1 male; mean age 56.0 ± 17.6) and 7 cases of follicular adenoma - FA (6 females, 1 male; mean age 49.4 ± 14.8) were also included in the study. The control group consisted of 24 cases of nodular goitre - NG (22 females, 2 males; mean age 48.9 ± 14.7) (Table 2).

**Table 1:** Sex, age, histopathological diagnosis of malignant thyroid tumours in the studied patients

Case number	Sex	Age	Histopathological diagnosis	TNM staging system	American Joint Committee on Cancer (AJCC) grouping system
1	F	23	PTC, classic variant	pT1bNxM0	I

2	F	38	PTC, classic variant	pT1N0M0	I

3	F	52	PTC, classic variant	pT2N0M0	II

4	F	71	PTC, classic variant	pT2N0Mx	II

5	F	47	PTC, classic variant	pT1aN0M0	I

6	F	24	PTC, classic variant	pT1N0Mx	I

7	F	33	PTC, classic variant	pT2aNxMx	II

8	F	28	PTC, classic variant	pT1bNxMx	I

9	F	40	PTC, classic variant	pT4N0Mx	IVA

10	F	17	PTC, classic variant	pT2bN1aM0	III

11	M	47	PTC, classic variant	pT2aN1aM0	III

12	M	31	PTC, classic variant	pT2aN0M0	II

13	F	16	PTC, classic variant	pT2aN0M0	II

14	F	61	PTC, classic variant	pT2aN0M0	II

15	F	48	PTC, classic variant	pT4N1aM0	IVA

16	M	30	PTC, classic variant	pT4N1bM0	IV

17	F	31	PTC, classic variant	pT1NxMx	I

18	F	40	PTC, classic variant	pT1aNxMx	I

19	F	28	PTC, classic variant	pT1aNxMx	I

20	F	25	PTC, classic variant	pT1aN1aMx	I

21	F	49	PTC, tall-cell variant	pT2bN0M0	II

22	F	52	PTC, tall-cell variant	pT4N0M0	IVA

23	F	59	PTC, tall-cell variant	pT4bN1Mx	IV

24	F	36	PTC, follicular variant	pT2aNxMx	II

25	M	28	PTC, follicular variant	pT2N1aMx	II

26	M	52	PTC, follicular variant	pT3NxMx	III

27	F	62	PTC, follicular variant	pT1aNxMx	I

28	F	26	PTC, follicular variant	pT4N1aM0	IVA

29	F	56	PTC, follicular variant	pT3aN0M0	III

30	F	68	PTC, follicular variant	pT2bN1aM0	II

31	M	66	PTC, follicular variant	pT4aN0M0	IVA

32	F	55	PTC, follicular variant	pT2aN0M0	II

33	M	74	MTC	pT4N0M0	IVA

34	F	32	MTC	pT1NxMx	I

35	F	32	MTC	pT1NxMx	I

36	F	59	MTC	pT3N1bM0	III

37	F	56	MTC	pT3N0M0	III

38	F	71	MTC	pT4N1aM0	IVA

39	F	68	MTC	pT3aN0M0	III

**Table 2:** Sex, age, histopathological diagnosis of benign thyroid lesions in the studied patients.

Case number	Sex	Age	Histopathological diagnosis
1	F	49	FA

2	F	52	FA

3	M	75	FA

4	F	29	FA

5	F	41	FA

6	F	41	FA

7	F	59	FA

8	F	40	NG

9	F	42	NG

10	F	30	NG

11	F	48	NG

12	F	42	NG

13	F	64	NG

14	F	69	NG

15	F	72	NG

16	F	50	NG

17	F	51	NG

18	F	63	NG

19	F	49	NG

20	F	49	NG

21	F	38	NG

22	M	52	NG

23	F	31	NG

24	F	29	NG

25	M	75	NG

26	F	28	NG

27	F	30	NG

28	F	72	NG

29	F	50	NG

30	F	40	NG

31	F	59	NG

The whole study cohort comprised 70 persons: 60 females and 10 males, the mean age was 46.4 ± 15.8 years (age ranged from 16 to 75 years). Histopathological diagnoses for malignant thyroid lesions, according to WHO Classification of Tumours [14], have been obtained from pathomorphological reports and are presented in Table 1, together with TNM classification and AJCC stage groupings [15].

In the study design, macroscopically unchanged thyroid tissue served for the reference standard (calibrator).

### Isolation of total RNA and reverse transcription (RT)

Total RNA was extracted from fresh tisssues, using RNeasy Protect Midi Kit, (Qiagen, Hilden, Germany), according to the manufacturer's recommendations. RNA concentration was spectrophotometrically assessed by measuring absorbance at 260 and 280 nm (Ultraspec 2000 UV/Visible Spectrophotometer, Pharmacia Biotech, Sweden).

Afterwards the reverse transcription (RT) was performed in a Personal Thermocycler (Eppendorf, Germany), using 1 μg of total RNA in the presence of 50 μM of oligo d(T)16 and Reverse Transcriptase MultiScribe™ (50 U/μl) in a total volume of 30 μl, including also: 10× TaqMan RT Buffer (containing 100 mM Tris-HCL pH 8.3, 500 mM KCL), MgCl_2 _solution (25 mM), dNTPs mixture (10 mM), RNAse Inhibitor (20 U/μl), and nuclease-free water (TaqMan Reverse Transcriptase Reagents, Applied Biosystems, Foster City, CA, USA). The reactions were conducted for 10 minutes at 25°C, followed by 30 minutes at 48°C, afterwards the samples were heated for 5 min to 95°C, and next placed at 4°C. The cDNA concentration and purity were spectrophotometrically assessed by measuring absorbance at 260 and 280 nm (Ultraspec 2000 UV/Visible Spectrophotometer, Pharmacia Biotech, Sweden).

### Quantitative analysis of the relative amount of PIN1 mRNA by real-time PCR

An established Relative Quantification Polymerase Chain Reaction (RQ-PCR) assay for PIN1 mRNA expression was conducted in ABI PRISM 7500 Sequence Detection System (Applied Biosystems), according to the manufacturer's protocol. The PCR reactions for *PIN1 *gene were run with 50 ng of cDNA in a total volume of 50 μl, using TaqMan^® ^Universal PCR Master Mix (Applied Biosystems, Foster City, CA, USA) and the predesigned and labelled primer/probe set (Assays-on-Demand™ Gene Expression assay mix, Hs01598309_ml, Applied Biosystems). After initial incubation at 50°C for 2 min to allow uracil-N-glycosylase (UNG) digestion, and at 95°C for 10 min to activate the AmpliTaq Gold^®^DNA polymerase, both of them were provided by the Universal PCR Master Mix, the samples were amplified through 40 biphasic cycles of 95°C for 15 sec and 60°C for 1 min (Table 3).

**Table 3:** Real-time PCR conditions for *PIN1 *gene amplification.

Times and temperatures			
**Initial setup**		**Each of 40 cycles**	
		
		**Denaturation**	**Annealing/Elongation**

HOLD	HOLD	CYCLE	
		
UNG activation 2 min, 50°C	10 min, 95°C	15 s, 95°C	1 min, 60°C

Macroscopically unchanged thyroid tissue was used as a calibrator (standard sample). Amplification reactions were done in triplicate for each sample (cDNA from the same PCR reaction but in separate wells). Controls with no template cDNA were used with each assay (negative control).

The expression levels of *β-actin *gene (*ACTB*) were measured, as endogenous control (reference gene), using the appropriate Assays-on-Demand™ Gene Expression product (Hs99999903_ml, Applied Biosystems Foster City, CA, USA).

Both gene expressions were measured for each thyroid lesion sample in the same PCR reaction but in separate wells.

Assays-on-Demand™ Gene Expression product consists of 20 × mix of unlabelled PCR primers (18 μM for each) and TaqMan^® ^MGB probes (5 μM) with FAM™ (6-carboxy-fluorescein) at the 5' end as the reporter dye, and a non-fluorescent quencher (TAMRA, 6-carboxy-tetramethylrhodamine) at the 3' end. The two-minute, 50°C step was required for optimal AmpErase^® ^UNG activity when TaqMan^® ^Universal PCR Master Mix (P/N 4304437) was used.

The fluorescence signal was measured in real-time PCR in the extension phase of the PCR reaction, and the measurement, proportional to the quantity of sample cDNA in the reaction, was plotted as an amplification curve against cycle number. A threshold value of fluorescence in the exponential part of the amplification curve was selected, and - for each sample - the number of cycles was measured, which was needed by the signal to reach the threshold (threshold cycle, C_T_). The larger were the quantities of the starting material, the lower were C_T _values.

### Data analysis

Data analysis of relative gene expression was performed with the Taq-Man SDS analysis software on an ABI PRISM 7500 Sequence Detection System (Applied Biosystems), and the results were exported to Excel sheets for further processing.

Fluorescence emission data were determined as C_T _values for each reaction and, for each sample, triplicate C_T _values were averaged. The average C_T _value for *β-actin *was subtracted from the average *PIN1 *C_T _value to yield the ΔC_T _value (ΔC_T _= C_T _target - C_T _reference). Normalization to the reference gene (*β-actin*) has been necessary to account for the variabilities in sample quantity and quality, and for the variabilities in PCR efficiency among samples. The assay, described in this report, involves the determination of a ΔΔC_T _value. This is calculated by: ΔΔC_T _= ΔC_T _test sample - ΔC_T _calibrator sample. The higher is the ΔΔC_T _value, the lower is the expression of *PIN1 *in the specimen. Fold-differences, representing relative expression results, are calculated using the following equation: relative fold increase = 2^-ΔΔC^T. In order to evaluate the analytical sensitivity, specificity, and accuracy of the assay, serial dilutions were performed.

### Statistical analysis

The data were statistically analyzed, using Newman-Keuls' multiple comparison test followed by Kruskal-Wallis' test, in order to compare the level of expression values (RQ) among the four studied independent groups (PTC, MTC, FA, NG). Basic measures of location (i.e. mean, median), measures of dispersion (SD, SEM), and minimum and maximum values were calculated to provide detailed descriptions of gene expressions in selected groups. Additionally, box-and-whiskers plots were introduced to display the difference in gene expressions levels in different samples.

The Kruskal-Wallis' test was used to compare the level of expression values (RQ) among three variants of PTC (PTC v. classic, PTC v. tall-cell, PTC v. follicular). The Spearman's rank correlation coefficient and U Mann-Whitney's test were performed in order to correlate the level of expression of *PIN1 *gene (RQ values) with examined parameters (age, sex, tumour size, classified according to TNM definition of primary tumours - pT1, pT2, pT3, pT4).

The accepted level of statistical significance was at p < 0.05. The results are presented as means ± SEM and means ± SD values.

Statistica for Windows 7.0 program was applied for calculations.

## Results

The specimens were amplified in the ABI PRISM 7500 Sequence Detection System in reaction, containing primers and probes for *PIN1 *gene and a control gene, *β-actin*. The Sequence Detection System software, provided with the instrument, analyses the fluorescence data, generated during the reaction, and calculates the cycle number at which fluorescence crosses the threshold value (C_T_). Triplicate amplifications of the sample produced nearly identical, overlapping curves, from which C_T _values were calculated.

In our experiment low C_T _values for *PIN1 *gene in all malignant thyroid tissues (PTC - 32 and MTC - 7 specimens) were observed.

The assay, described in this paper, was based on the determination of a ΔΔC_T _value (ΔΔC_T _method) for each sample (the greater was the ΔΔC_T _value, the lower was the expression of *PIN1 *gene) and on calculating the difference in *PIN1 *expression level between tumour sample and calibrator (RQ value). The results for calculated RQ in the studied groups (PTC, MTC, FA, NG) are presented in Table 4. The RQ value for PTC, classic variant, ranged from 0.441 to 52.279 (the mean RQ was 8.43); for PTC, follicular variant, ranged from 0.804 to 9.124 (the mean RQ was 3.03) and for PTC, tall-cell variant, ranged from 0.363 to 12.616 (the mean RQ was 6.42).

**Table 4:** The expression level (RQ) of *PIN1 *gene, calculated by the ΔΔC_T _method in the studied groups.

Group	No. of cases	Mean RQ	Range of RQ values
PTC	32	6.72	0.363-52.279

MTC	7	5.55	0.917-19.408

FA	7	2.79	0.511-8.207

NG	24	2.15	0.322-13.153

**Figure 1:**
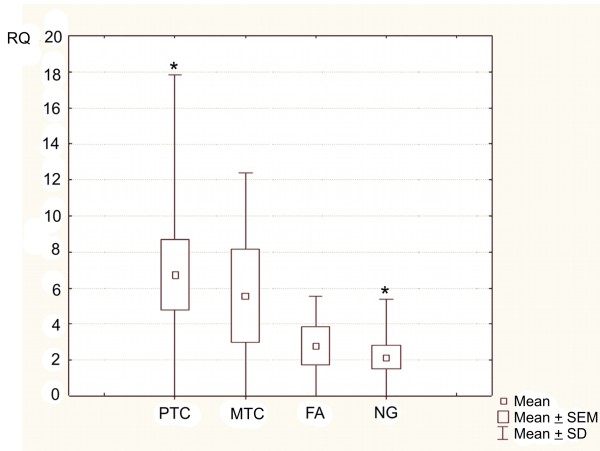
**Box-and-whisker plots, representing the expression of *PIN1* gene in the studied groups (PTC, MTC, FA, NG)**. Results are calculated as RQ values. Whiskers represent means ± SD (standard deviation) for particular groups. Boxes represent means ± SEM (standard error of mean). The data were statistically analyzed, using Newman-Keuls' multiple comparison test, followed by Kruskal-Wallis' test, in order to compare the level of expression values (RQ) among the four studied independent groups, p < 0.05; * - p < 0.031977 (Neuman-Keuls' test).

**Figure 2:**
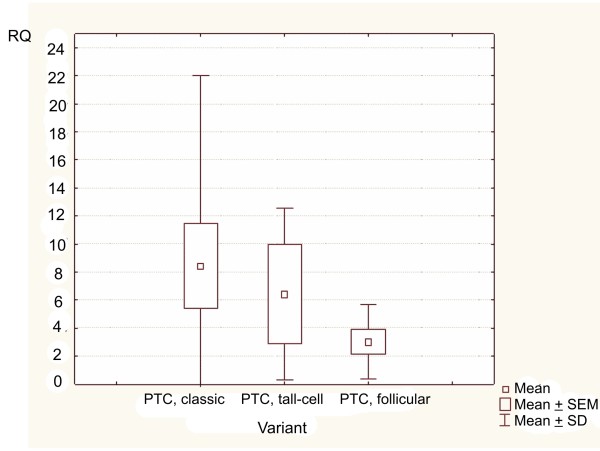
**Box-and-whisker plots, representing the difference in the expression of *PIN1* gene in the variants of PTC (PTC v. classic, PTC v. tall-cell, PTC v. follicular)**. Results are calculated as RQ values; no significant differences among studied groups are shown. Whiskers represent means ± SD (standard deviation) for particular groups. Boxes represent means ± SEM (standard error of mean). The results were statistically analyzed using Kruskal-Wallis' test, p = 0.9501.

**Figure 3:**
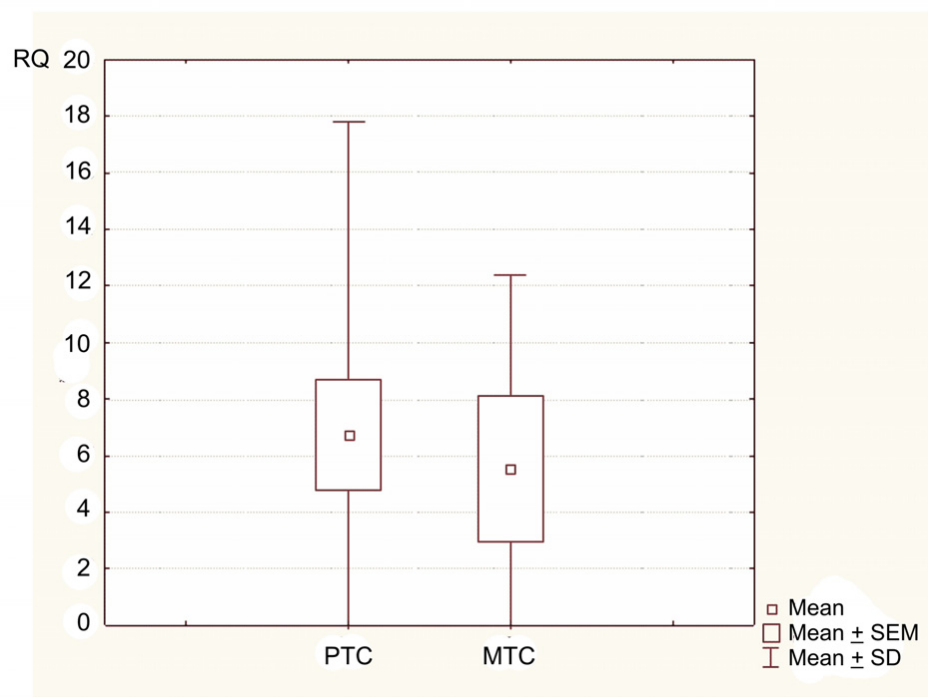
**Box-and-whisker plots, representing the difference in the expression of *PIN1* gene between the PTC group and the MTC group**. Results are calculated as RQ values; no significant differences between studied groups are demonstrated. Whiskers represent means ± SD (standard deviation) for particular groups. Boxes represent means ± SEM (standard error of mean). The results were statistically analyzed using Mann-Whitney's U test, p = 0.6873.

Kruskal-Wallis' test, applied for RQ comparison between the four studied groups (PTC, MTC, FA, NG), showed statistically significant differences (p = 0.0233) among them (Figure 1). The results of Newman-Keuls' multiple comparison test revealed statistically significant differences in RQ values only between the PTC group (higher RQ values) and the control group - NG (p = 0.031977). In cases of other groups, the differences in RQ values were not significant (p > 0.05) (Table 5). Similarly, the results of Kruskal-Wallis' test did not show any significant differences among the particular variants of PTC (PTC v. classic, PTC v. tall-cell, PTC v. follicular) (p = 0.9501) (Figure 2). Additionally, in the whole studied group of PTCs, no correlations were found between *PIN1 *expression level and patients' sex, age or tumour size (p > 0.05). The results of Mann-Whitney's U test confirmed no significant differences in RQ values between the PTC group and MTC group (p = 0.6873) (Figure 3).

**Table 5:** Statistical analysis of results - comparison of RQ value among the four studied groups (PTC, MTC, FA, NG). Newman-Keuls' test, p - level of significance.

	PTC	MTC	FA	NG
**PTC**		1.000000	1.000000	**0.031977**

**MTC**	1.000000		1.000000	0.154248

**FA**	1.000000	1.000000		1.000000

**NG**	**0.031977**	0.154248	1.000000	

## Discussion

The molecular pathogenesis of thyroid cancer is very complex and still unclear. Recent molecular studies on thyroid carcinogenesis have proved that many specific genetic alterations in protooncogenes are associated with development of thyroid cancer [16,17]. The mutational activation of MAPK/ERK signalling pathway is crucial to understand the carcinogenesis in the thyroid. The mutations of genes coding effectors of MAPK/ERK pathway (*RET*, *RAS*, *NTRK1*, *BRAF*) which rarely overlap, may be identified in more than 70% of PTC cases [18-20]. Additionally, the chromosomal rearrangements (*RET/PTC *or *Trk*) appear to be of crucial importance in activation of MAPK/ERK pathway [21,22].

Despite the fact that constitutive activation of the above mentioned pathway is a very important for the development of thyroid cancer, many other molecular regulatory mechanisms, involved in thyroid cancer transformation, are recognized.

In our study, we have examined whether the *PIN1 *gene participates in thyroid carcinogenesis.

The phosphorylation of proteins on serine or threonine residues preceding proline (pSer/Thr-Pro motifs), catalized by the peptidyl-prolyl *cis/trans *isomerase Pin1, has been confirmed as a major cell transformation mechanism activating different oncogenic pathways in many types of human tumours (including thyroid cancer) [9,13,23,24]. In immunohistochemical studies of thyroid tumours, as well as of other types of cancers (e.g., oral squamous carcinoma), high Pin1 expression promotes cyclin D1 overexpression, directly or through accumulation of beta-catenin [13,25]. It has been reported that *PIN1 *expression is regulated by the transcription factor E2F and Neu/Ras signalling. The enhanced expression and stability of cyclin D1 by transcriptional activation and/or posttranslational stabilization by *PIN1 *gene or Pin1 protein may lead to cell proliferation and transformation. Therefore, *PIN1 *might be a crucial factor in modulating upregulation of cyclin D1 level by *Neu/Ras *oncogenic signalling [7,26].

Bearing in mind all these facts, in our present study, we have assessed *PIN1 *gene expression level in benign and malignant thyroid lesions. In our earlier study, we demonstrated that *cyclin D1 *gene expression levels were higher in malignant thyroid tumours (PTC, MTC), when compared to those in NG or FA groups [27]. Our present results have revealed statistically significant differences in expression of PIN1 mRNA between PTC group and benign thyroid lesions (FA, NG) (higher RQ value in PTC). It should be stressed that our data are in accordance with observations of other authors [13]. Nakashima et al. [13] have observed overexpression of cyclin D1 mRNA in PTC (thyroid tumours from a radiocontaminated area), taking course with high expression of *PIN1 *gene. In the quoted paper, correlation between high Pin1 protein level and cyclin D1 immunoexpression level in PTC has also been confirmed [13]. It is important that Pin1 can promote cyclin D1 overexpression directly or through beta-catenin stabilization [6,13]. Our present results, which have focused on PIN1 mRNA expression, as well as our earlier data, concerning cyclin D1 mRNA overexpression in thyroid malignancy, may suggest that one of the major regulatory mechanisms in cell transformation in thyroid carcinogenesis is *PIN1 *gene activity [27]. It is tempting to speculate that *PIN1 *gene positively regulates *cyclin D1 *gene function not only through posttranslational stabilization but also by an effect on transcriptional level.

In our present study, we have applied real-time relative quantification PCR assay for PIN1 mRNA expression and, employing this extremely sensitive and precise method, we have confirmed overexpression of *PIN1 *gene in PTC. These results may suggest a diagnostic utility of PIN1 mRNA expression - as a malignant lesion marker - for thyroid PTC. However, the results should be confirmed in larger number of PTC cases. The utility of *PIN1 *gene may be - in future - very crucial for diagnostics and treatment of thyroid cancer (especially of PTC), based on the facts that overexpression of Pin1 has been found to be an excellent prognostic marker in some other human cancers (e.g., prostate, breast, colorectal cancers) [8,10,23]. In these immunohistochemical studies, strong relationship between Pin1 protein level and clinical outcome has been observed [8,10,23]. Unfortunately, it is difficult to discuss the relationship between PIN1 mRNA and prognosis in PTC, because - according to our knowledge - there were no reported studies, focusing on the evaluation of *PIN1 *gene expression in association with pathological features and clinical outcome of PTC, so far. Our present data have not documented any significant differences in the expression level of PIN1 mRNA among the particular variants of PTC (classic vs. tall-cell, vs. follicular variants). Moreover, no correlations were found between *PIN1 *expression level and patients' sex, age or tumour size in PTC group.

In summary, *PIN1 *gene expression may have - in future - certain significance in PTC diagnostics and in understanding its pathogenesis. However, our results - due to the small number of cases - do not yet allow considering *PIN1 *gene as a prognostic molecular PTC marker.

## Competing interests

The authors declare that they have no competing interests.

## Authors' contributions

AL designed and coordinated the study and wrote the manuscript; EB and KC carried out the molecular genetic studies; JL participated in performing molecular studies; WK coordinated the collection of tissue samples; AC-M participated in performing molecular studies and drafted the manuscript.

## References

[B1] Joseph JD Yeh ES Swenson KI Means AR Winkler KE The peptidyl-prolyl isomerase Pin1 Prog Cell Cycle Res 2003 5 477 487 14593743

[B2] Campbell HD Webb GC Fountain S Young IG The human PIN1 peptidyl-prolyl cis/trans isomerase gene maps to human chromosome 19p13 and the closely related PIN1L gene to 1p31 Genomics 1997 44 157 162 10.1006/geno.1997.4854 9299231

[B3] Stukenberg PT Kirschner MW Pin1 acts catalytically to promote a conformational change in Cdc 25 Mol Cell 2001 7 1071 1083 10.1016/S1097-2765(01)00245-3 11389853

[B4] Wulf GM Ryo A Wulf GG Lee SW Niu T Petkova V Lu KP Pin1 is overexpressed in breast cancer and cooperates with Ras signaling in increasing the transcriptional activity of c-Jun towards cyclin D1 EMBO J 2001 20 3459 3472 10.1093/emboj/20.13.3459 11432833 PMC125530

[B5] Wulf GM Liou YC Ryo A Lee SW Lu KP Role of Pin1 in the regulation of p53 stability and p21 transactivation, and cell cycle checkpoints in response to DNA damage J Biol Chem 2002 277 47976 47979 10.1074/jbc.C200538200 12388558

[B6] Ryo A Nakamura M Wulf G Liou YC Lu KP Pin1 regulates turnover and subcellular localization of beta-catenin by inhibiting its interaction with APC Nat Cell Biol 2001 3 793 801 10.1038/ncb0901-793 11533658

[B7] Ryo A Liou YC Wulf G Nakamura M Lee SW Lu KP PIN1 is an E2F target gene essential for Neu/Ras-induced transformation of mammary epithelial cells Mol Cell Biol 2002 22 5281 5295 10.1128/MCB.22.15.5281-5295.2002 12101225 PMC133940

[B8] Ayala G Wang D Wulf G Frolov A Li R Sowadski J Wheeler TM Lu KP Bao L The prolyl isomerase Pin1 is a novel prognostic marker in human prostate cancer Cancer Res 2003 63 6244 6251 14559810

[B9] Bao L Kimzey A Sauter G Sowadski JM Lu KP Wang DG Prevalent overexpression of prolyl isomerase Pin1 in human cancers Am J Pathol 2004 164 1727 1737 15111319 10.1016/S0002-9440(10)63731-5 PMC1615639

[B10] Wulf G Ryo A Liou YC Lu KP The prolyl isomerase Pin1 in breast development and cancer Breast Cancer Res 2003 5 76 82 10.1186/bcr572 12631385 PMC154150

[B11] Rippmann JF Hobbie S Daiber C Guillard B Bauer M Birk J Nar H Garin-Chesa P Rettig WJ Schnapp A Phosphorylation-dependent proline isomerization catalyzed by Pin1 is essential for tumor cell survival and entry into mitosis Cell Growth Differ 2000 11 409 416 10939594

[B12] Takahashi K Uchida C Shin RW Shimazaki K Uchida T Prolyl isomerase, Pin1: new findings of post-translational modifications and physiological substrates in cancer, asthma and Alzheimer's disease Cell Mol Life Sci 2008 65 359 375 10.1007/s00018-007-7270-0 17965833 PMC11131890

[B13] Nakashima M Meirmanov S Naruke Y Kondo H Saenko V Rogounovitch T Shimizu-Yoshida Y Takamura N Namba H Ito M Abrosimov A Lushnikov E Roumiantsev P Tsyb A Yamashita S Sekine I Cyclin D1 overexpression in thyroid tumours from a radio-contaminated area and its correlation with Pin1 and aberrant beta-catenin expression J Pathol 2004 202 446 455 10.1002/path.1534 15095272

[B14] DeLellis RA Lloyd RV Heitz PU Eng C (eds) World Health Organization classification of tumours: Pathology and genetics of tumours of endocrine organs 2004 IARC Press, Lyon

[B15] American Joint Committee on Cancer Thyroid AJCC Cancer Staging Manual 2002 6 New York, NY: Springer 77 87

[B16] Lewiński A Ferenc T Sporny S Jarząb B Thyroid carcinoma: diagnostic and therapeutic approach; genetic background Endocr Regul 2000 34 99 113 10991553

[B17] Lewiński A Wojciechowska K Genetic background of carcinogenesis in the thyroid gland Neuroendocrinol Lett 2007 28 77 105 17435680

[B18] Kimura ET Nikiforova MN Zhu Z Knauf JA Nikiforov YE Fagin JA High prevalence of BRAF mutations in thyroid cancer: genetic evidence for constitutive activation of the RET/PTC-RAS-BRAF signaling pathway in papillary thyroid carcinoma Cancer Res 2003 63 1454 1457 12670889

[B19] Soares P Trovisco V Rocha AS Lima J Castro P Preto A Maximo V Botelho T Seruca R Sobrinho-Simoes M BRAF mutations and RET/PTC rearrangements are alternative events in the pathogenesis of PTC Oncogene 2003 22 4578 4580 10.1038/sj.onc.1206706 12881714

[B20] Frattini M Ferrario C Bressan P Balestra D De Cecco L Mondellini P Bongarzone I Collini P Gariboldi M Pilotti S Pierotti MA Greco A Alternative mutations of BRAF, RET, and NTRK1 are associated with similar but distinct gene expression patterns in papillary thyroid cancer Oncogene 2004 23 7436 7440 10.1038/sj.onc.1207980 15273715

[B21] Santoro M Melillo RM Fusco A RET/PTC activation in papillary thyroid carcinoma: European Journal of Endocrinology Prize Lecture Eur J Endocrinol 2006 155 645 653 10.1530/eje.1.02289 17062879

[B22] Brzeziańska E Karbownik M Migdalska-Sęk M Pastuszak-Lewandoska D Włoch J Lewiński A Molecular analysis of the RET and NTRK1 gene rearrangements in papillary thyroid carcinoma in the Polish population Mutat Res 2006 599 26 35 16483615 10.1016/j.mrfmmm.2005.12.013

[B23] Kuramochi J Arai T Ikeda S Kumagai J Uetake H Sugihara K High Pin1 expression is associated with tumor progression in colorectal cancer J Surg Oncol 2006 94 155 160 10.1002/jso.20510 16847925

[B24] Zhou CX Gao Y Aberrant expression of beta-catenin, Pin1 and cyclin D1 in salivary adenoid cystic adenoma: relation to tumor proliferation and metastasis Oncol Rep 2006 16 505 511 16865250

[B25] Leung KW Tsai CH Hsiao M Tseng CJ Ger LP Lee KH Lu PJ Pin1 overexpression is associated with poor differentiation and survival in oral squamous cell carcinoma Oncol Rep 2009 21 1097 1104 19288014 10.3892/or_00000329

[B26] Liou YC Ryo A Huang HK Lu PJ Bronson R Fujimori F Uchida T Hunter T Lu KP Loss of Pin1 function in the mouse causes phenotypes resembling cyclin D1-null phenotypes Proc Natl Acad Sci USA 2002 99 1335 1340 10.1073/pnas.032404099 11805292 PMC122191

[B27] Brzeziańska E Cyniak-Magierska A Sporny S Pastuszak-Lewandoska D Lewiński A Assessment of *cyclin D1 *gene expression as a prognostic factor in benign and malignant thyroid lesions Neuroendocrinol Lett 2007 28 341 350 17693985

